# Effectiveness of Fiber Reinforcement on the Mechanical Properties and Shrinkage Cracking of Recycled Fine Aggregate Concrete

**DOI:** 10.3390/ma9030131

**Published:** 2016-02-26

**Authors:** Jeongsoo Nam, Gyuyong Kim, Jaechul Yoo, Gyeongcheol Choe, Hongseop Kim, Hyeonggil Choi, Youngduck Kim

**Affiliations:** 1 Structure Engineering Research Center, Tokyo Institute of Technology, Yokohama 226-8503, Japan; nam.j.aa@m.titech.ac.jp; 2 Department of Architectural Engineering, Chungnam National University, Daejeon 34134, Korea; 52jc@cnu.ac.kr (J.Y.); choegc@cnu.ac.kr (G.C.); hskim87@cnu.ac.kr (H.K.); 3 Graduate School of Engineering, Muroran Institute of Technology, Muroran 050-8585, Japan; chg810@mmm.muroran-it.ac.jp; 4 Offshore Plant Research Division, Korea Research Institute of Ship and Ocean Engineering, Daejeon 34103, Korea; kyd000@kriso.re.kr

**Keywords:** recycled fine aggregate, fiber-reinforced concrete (FRC), small fiber volume fractions, mechanical properties, shrinkage cracking

## Abstract

This paper presents an experimental study conducted to investigate the effect of fiber reinforcement on the mechanical properties and shrinkage cracking of recycled fine aggregate concrete (RFAC) with two types of fiber—polyvinyl alcohol (PVA) and nylon. A small fiber volume fraction, such as 0.05% or 0.1%, in RFAC with polyvinyl alcohol or nylon fibers was used for optimum efficiency in minimum quantity. Additionally, to make a comparative evaluation of the mechanical properties and shrinkage cracking, we examined natural fine aggregate concrete as well. The test results revealed that the addition of fibers and fine aggregates plays an important role in improving the mechanical performance of the investigated concrete specimens as well as controlling their cracking behavior. The mechanical properties such as compressive strength, splitting tensile strength, and flexural strength of fiber-reinforced RFAC were slightly better than those of non-fiber-reinforced RFAC. The shrinkage cracking behavior was examined using plat-ring-type and slab-type tests. The fiber-reinforced RFAC showed a greater reduction in the surface cracks than non-fiber-reinforced concrete. The addition of fibers at a small volume fraction in RFAC is more effective for drying shrinkage cracks than for improving mechanical performance.

## 1. Introduction

Concrete is the most widely used material among construction materials due to its superior strength and durability [1]. In Korea, over the past few decades, concrete has contributed significantly to national development and economic growth, and it has been employed actively in the construction of social infrastructure facilities and private residences. However, when a large amount of concrete is used, the treatment of construction wastes must be considered [2,3,4].

According to the Ministry of Environment report in Korea, in 2012, the amount of construction waste was 186,629 tons per day, which is a significant amount that has been increasing every year. The contribution of concrete waste is the largest among all other construction wastes [5]. The recycling of waste concrete is therefore essential for realizing a sustainable construction industry, and studies on the recycling of waste concrete have attracted global interest. Most of these studies focused on reusing aggregates manufactured from waste concrete.

Many researchers have attempted to ensure adequate performance for recycled aggregate construction materials [6,7,8,9,10]. Zaharieva *et al.* [6] investigated the durability and mechanical properties of recycled aggregate concrete under freeze-thaw action. The durability and mechanical performance of a specimen with a replacement ratio of 100% of recycled coarse aggregate were lower than those of a specimen using the natural aggregate after freezing and thawing. Pedro *et al.* [7,8] studied the effects of recycled coarse aggregates that were obtained from different sources on the mechanical properties, durability and shrinkage of the recycled aggregate concrete. In their study, it was found that concrete containing the recycled coarse aggregate subjected to the primary and secondary crushing process showed superior performance to the concrete subjected to only the primary crushing process; this was attributed to efficient removal of mortar by the primary and secondary crushing process. However, the durability and shrinkage resistance of recycled coarse aggregate concrete were less than those of natural coarse aggregate concrete. It is worth noting that various types of recycled aggregate have been used in construction materials over the past few decades; recycled aggregate is obtained from different sources. Hence, the mechanical properties and durability of recycled aggregate concrete depend on the quality of the recycled aggregates used in the concrete. The recycled aggregates used in construction materials are affected by the quality standards of each country; generally, recycled aggregates do not perform as well as natural aggregates. In particular, in a previous research [11,12,13,14], recycled aggregate concrete was shown to have lower strength and larger drying shrinkage compared to natural aggregate concrete. This behavior is attributed to the influence of the characteristics of coarse and fine recycled aggregate, such as higher absorption and impurity content [15,16]. The large drying shrinkage of recycled aggregate concrete inhibits strength development and increases cracks. Chemical admixtures or reinforcing fibers have been considered for use with recycled aggregate concrete to mitigate the shortcomings of recycled aggregate concrete.

Although there have been studies on recycled aggregate concrete, studies focusing on the performance improvement of fiber-reinforced recycled aggregate concrete have been very limited. Carneiro *et al.* [17] conducted experimental investigations on the mechanical properties of construction and demolition waste concrete with hooked-end steel fibers. Recycled aggregate concrete with hooked-end steel fibers exhibited a better mechanical performance with a 0.75% volume fraction than natural aggregate concrete. Akça *et al.* [18] investigated the flexural and splitting tensile strengths of recycled aggregate concrete with polypropylene fibers. From the experiments, both the flexural tensile strength and the splitting tensile strength increased by 1.0% and 1.5% fiber volume fraction, respectively. Mesbah and Buyle-Bodin [19] studied the efficiency of polypropylene and metallic fibers for controlling the shrinkage cracking in recycled aggregate mortars. The crack width of recycled aggregate mortar was less than that of natural aggregate mortar when polypropylene and metallic fibers with maximum volume fractions of 0.5% and 1.0%, respectively, were added. Richardson *et al.* [20] investigated the freeze/thaw durability of polypropylene fiber-reinforced concrete with recycled demolition aggregate. According to the results of the study, it is possible to suppress the reduction in compressive strength and mass loss of recycled aggregate concrete with 1.0% volume fraction of polypropylene fibers before and after 56 cycles of freeze-thaw.

Based on the above studies, it is clear that fiber reinforcement in recycled aggregate concrete plays an important role in improving the mechanical properties and durability as well as controlling the shrinkage cracking behavior. Nonetheless, investigations on the mechanical properties, durability, and shrinkage cracking of recycled aggregate concrete with fiber reinforcement are still very few. Furthermore, most of the studies investigated fiber-reinforced recycled aggregate concrete with a relatively large amount of fiber. However, to consider the economic efficiency of recycled aggregate concrete, it is necessary to minimize the increase in cost by using a small amount of fiber for fiber reinforcement. Therefore, the objective of the current study is to investigate experimentally the effectiveness of added fibers at small volume fractions on the mechanical properties and shrinkage cracking of recycled fine aggregate concrete (RFAC). Various characteristics of fiber-reinforced RFAC pertaining to mechanical properties and shrinkage cracking were investigated, including compressive strength, modulus of elasticity, splitting tensile strength, flexural strength, drying shrinkage, and surface cracking. The results obtained provide valuable information for enhancing the capacity of fiber-reinforced RFAC to withstand mechanical degradation and reduction in shrinkage cracking, particularly from the viewpoint of using recycled aggregate materials in the construction industry field.

## 2. Experimental Program

### 2.1. Materials

**Figure 1:**
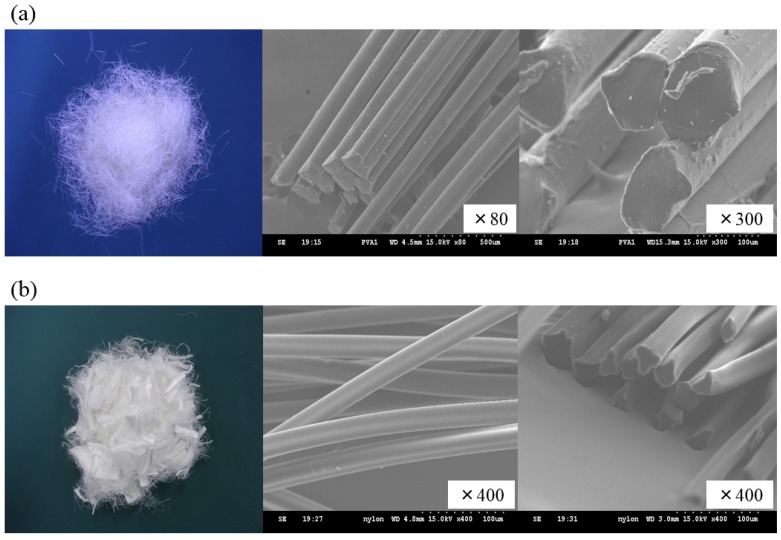
Fiber shapes: (**a**) polyvinyl alcohol (PVA) fiber; (**b**) nylon fiber.

The physical properties of the materials used are listed in Table 1, except those of recycled fine aggregate (RFA) and fibers. The used natural fine aggregate (NFA) met the requirements specified in ASTM C33 [21]. Table 2 lists the properties of RFA used in this study along with the requirements specified in KS F 2573 [22]. Most of the requirements in KS F 2573 are similar to those in ASTM C33. As shown in Table 2, RFA satisfied the requirements specified in KS F 2573. All the aggregates used in this study were prepared in saturated surface dry (SSD) conditions before adding cement. The properties of the added fibers used in fiber-reinforced RFAC are listed in Table 3. Figure 1 shows the shape of polyvinyl alcohol (PVA) and nylon fibers in macro and micro scales.

### 2.2. Specimens

Table 4 lists the mixture proportions of concrete specimens. Six types of mixture for test specimens were investigated. Each mixture was classified by fine aggregate, and the type of added fibers with different volume fractions. In this study, the test specimens were used for two purposes—for comparing the mechanical properties and cracking behaviors of RFAC with a replacement ratio of 100% for natural fine aggregate concrete (NFAC), and for investigating the effects of fiber reinforcement with a small volume fraction on the improvement of performance for non-fiber-reinforced RFAC. In Table 4, the specimen’s ID indicates the type of fine aggregate, type of added fibers, and fiber volume fraction, sequentially. N-0 is a specimen for comparing the mechanical properties and shrinkage cracking behavior of RFAC specimens with and without fiber reinforcement. R-0 is a plain specimen for fiber-reinforced RFAC specimens. The fiber volume fraction of fiber-reinforced RFAC specimens is 0.05% and 0.1% for both PVA and nylon fibers.

The procedure for mixing the concrete mixtures is as follows. First, the cement and coarse and fine aggregate were mixed sufficiently. Second, mixing water and superplasticizer (SP) were added to the mixtures and mixed for 1.5 min. Then, the PVA or nylon fibers were added to the concrete mixtures, and mixing was continued for 1.5 min. Finally, the fresh concrete mixtures were cast into cylindrical- and prismatic-shaped molds as well as plat-ring-type and slab-type molds. From each mixture, six 100 × 200 mm cylinders and six 100 × 100 × 400 mm prisms were prepared for the tests on mechanical properties and drying shrinkage. All the fresh concrete had similar flow properties before casting. All the specimens were demolded, being air-cured at 20 ± 3 °C and at 60% ± 5% relative humidity for 1 day and cured at 20 ± 3 °C in water for 27 days.

### 2.3. Testing Methods

The flowability of the fresh concrete was measured according to the ASTM C143 [23] standard test. The air content of fresh concrete was measured as defined by the ASTM C231 [24] standard test. The compressive strength was measured according to the ASTM C39 [25] standard test by using cylindrical concrete specimens with a diameter of 100 mm and a height of 200 mm. Experimental investigations of the splitting tensile strength and flexural strength of the concrete specimens were conducted as defined by the ASTM C496 [26] and ASTM C78 [27] standard tests, respectively. The drying shrinkage test was conducted immediately after demolding using an embedded strain gage.

**Figure 2:**
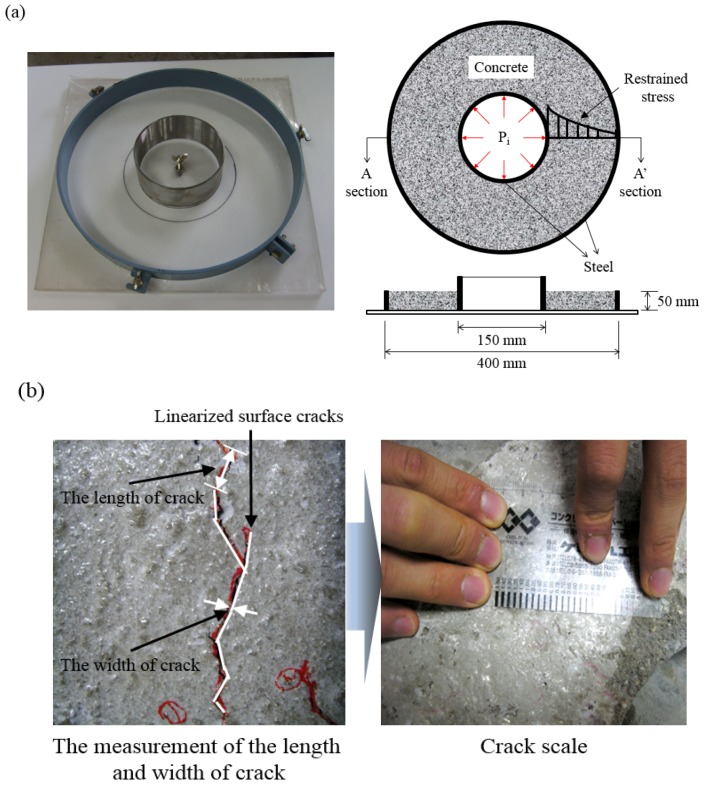
Overview of plat-ring-type test for investigation of shrinkage cracking of concrete [29]: (**a**) plat-ring-type test mold; (**b**) measurement method of the length and width of crack.

**Figure 3:**
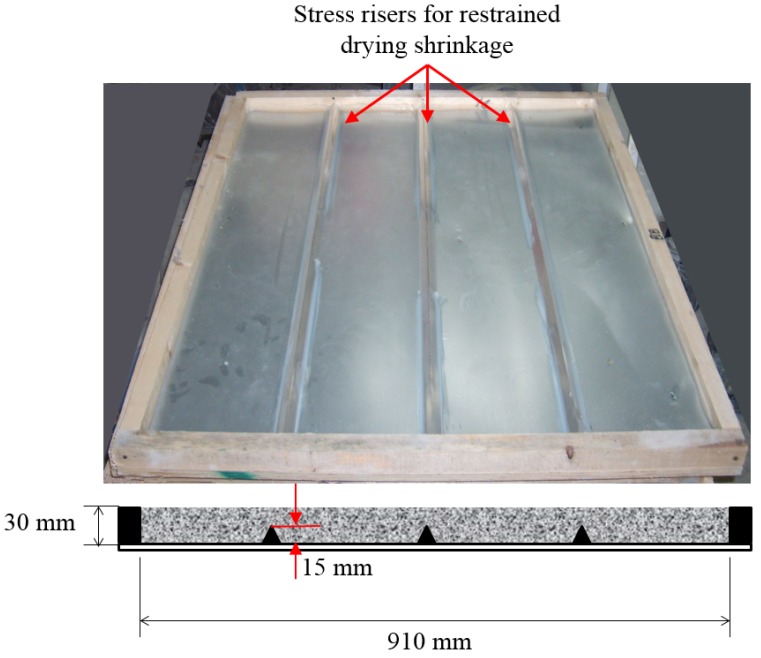
Slab-type test for evaluation of surface cracking of concrete.

To investigate the shrinkage cracking behavior of concrete specimens, plat-ring-type and slab-type tests were employed, as shown in Figure 2 and Figure 3, respectively. If the thickness and width of the specimens are thinner and wider than a standard test specimen in the ring and slab type test methods, it is not possible to confirm the time of cracking; however, the surface crack area of the concrete specimen can be obtained. Therefore, in this study, based on several preliminary shrinkage cracking tests, we applied plat-ring-type and slab-type test methods to quantitatively evaluate the crack reducing effect generated from the surface of concrete specimens. The plat-ring-type test was devised according to the ASTM C1581 [28] standard test as reference. To observe the amount of surface cracks in concrete caused by restrained drying shrinkage, the plat-ring-type test specimen is approximately three times thinner and approximately two times wider than the reference specimen. The reference specimen has a thickness of 150 ± 6 mm and width of 38 ± 3 mm. In previous research [29] on the plat-ring-type test of concrete, the restrained stress on the inner steel ring was larger than that of the outer steel ring, which affected the restrained shrinkage of concrete. Thus, the amount of surface cracks in a concrete specimen was dependent on the shrinkage capacity of concrete. To accelerate drying shrinkage, the plat-ring-type tests were carried out at a temperature of 36 ± 3 °C and at a relative humidity of 30% ± 10% in a drying chamber after casting concrete specimens. Also, we conducted a linearization of the unpatterned surface cracks in order to measure the length and width of cracks. The length and width of surface cracks of concrete specimens are measured by a crack scale for each linearized section.

In addition, the slab-type test was devised according to the ASTM C1579 [30] standard test as a reference. The slab-type test specimen is approximately three times thinner and approximately two times wider than the reference specimen. The reference specimen has a thickness of 100 ± 5 mm, length of 560 ± 10 mm, and width of 355 ± 10 mm. The surface cracking of concrete was induced by three stress risers with heights of 15 mm because the restrained stress is concentrated in the riser. Moreover, to confirm the behavior of long-term shrinkage cracking, the slab-type test was performed in outdoor environments over 16 weeks.

## 3. Results and Discussion

### 3.1. Properties of Fresh Concrete

Table 5 lists the properties of fresh concrete for each mixture. The flowability of all concrete mixtures achieved a target slump of 180 ± 20 mm with the addition of the superplasticizer. The target value range of slump in concrete mixtures was set based on the value obtained from N-0 and R-0. Fiber-reinforced RFAC mixtures were also adjusted such that the slump was within the target value range of slump set based on the non-fiber-reinforced concrete mixtures. When comparing the value of slump for N-0 and R-0 mixtures, it was observed that the flowability of R-0 was lower than that of N-0 for the same amount of added SP. This can be explained by the higher absorption ratio of recycled fine aggregate, which has quite a large absorption ratio compared to that of natural fine aggregate. The addition of PVA and nylon fibers to RFAC mixtures reduces their workability compared to R-0, with some differences arising depending on the type of added fibers and added SP. In the slump test results of fiber-reinforced RFAC mixtures, the slump value of R-PVA005 was higher than that of R-Ny005 at 0.05% fiber volume fraction; moreover, the slump value of R-Ny01 was higher than that of R-PVA01 at a range of 0.1% fiber volume fraction. It was intended with these results to show that the slump value of concrete mixtures depends on the added amount of SP. Comparing R-PVA01 and R-Ny005 under the same added amount of SP, although R-PVA01 has a large volume fraction compared to R-Ny005 it has a higher slump value than R-Ny005. The reason for this result can be explained by the properties of the fibers, such as diameter and density. Since PVA fibers have a larger diameter and density than nylon fibers, the number of added PVA fibers is less than that of nylon fibers. It can be inferred that PVA fibers have little effect on the workability. The air content of R-0 slightly increased compared to that of N-0; however, this was not a significant result. Moreover, the air content increased with the addition of fibers because fiber reinforcement can cause faulty mixing in fresh concrete [18]. A large amount of SP was added to the fiber-reinforced RFAC mixtures to reach the target slump; the amount of SP added to non-fiber-reinforced concrete mixtures was less compared to that added to the fiber-reinforced RFAC mixtures.

### 3.2. Compressive Strength

**Figure 4:**
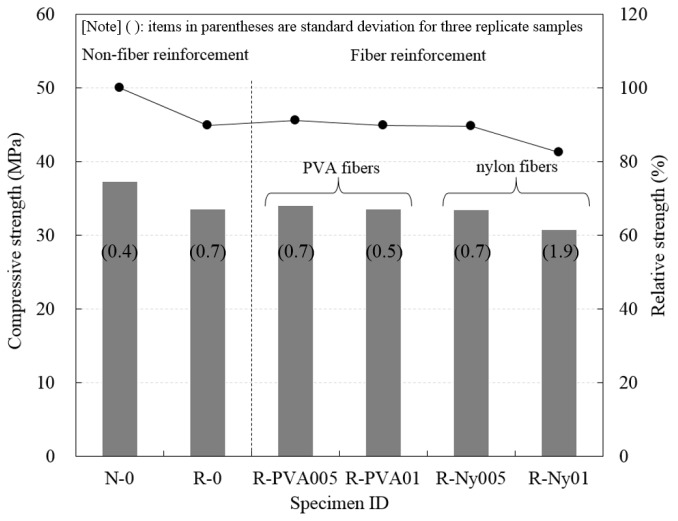
Compressive strength characteristics of concrete specimens at 28 days.

To investigate compressive strength of concretes, three replicate samples were tested. The results of compressive strength tests are shown in Figure 4. For the non-fiber-reinforced concrete specimens, the compressive strength of R-0 was lower than that of N-0. In previous researches [14,31,32], the reason for this behavior was explained by the influence of the weak mechanical bonding between recycled aggregate and cement paste. It is believed that recycled fine aggregate has an impurity content of 0.07% in contrast with natural fine aggregate; this inhibits strength development and effective mechanical bonding in R-0 specimens. The values of compressive strength of the fiber reinforced concretes were not found to be significantly different from the value of compressive strength of non-reinforced RFAC. Similar observations were previously reported by Jiang *et al.* [33] and Borg *et al.* [34]. The addition of fibers had no influence on the compressive strength of concrete specimens. With regard to the compressive strength of fiber-reinforced RFAC, the compressive strength of R-Ny01 was slightly lower than that of other specimens. It can be inferred that the higher air content of R-Ny01 in the fresh mixture has an effect on the reduction in compressive strength.

Additionally, the results exhibited that the relative strength of R-0 was lower than N-0 by 10.1%; further, in cases of fiber-reinforced RFAC, the relative strength of all specimens was lower than N-0, from 8.8% up to 17.5%. Khatib [14] reported that the relative compressive strength of concrete that had a 100% replacement ratio by recycled fine aggregate was significantly reduced compared to concrete with 25%, 50%, and 75% replacement ratios. In 100% replacement specimens, the reduction in relative strength at long-term aging was improved. In previous studies [31,32], the strength enhancement of RFAC was attributed to the possibility of a non-hydrated cement reaction in recycled fine aggregate at long-term aging. The effect of fiber reinforcement with a small volume fraction on the compressive strength of fiber-reinforced RFAC was not observed.

### 3.3. Modulus of Elasticity

**Figure 5:**
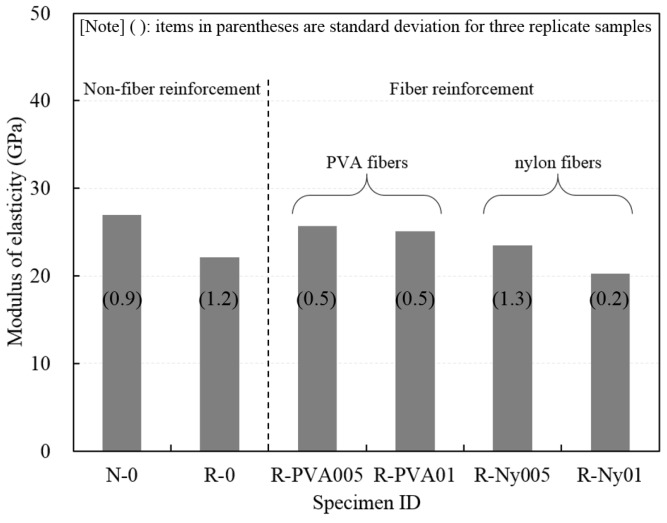
Modulus of elasticity of concrete specimens.

For each concrete mixture, three replicate samples were tested 28 days after casting. The mean modulus of elasticity values for concrete specimens are shown in Figure 5. The modulus of elasticity of all RFAC specimens was less than that of N-0. Evangelista and de Brito [31] reported that the reduction in the modulus of elasticity of RFAC was highly dependent on the replacement ratio by recycled fine aggregate; when comparing the modulus of elasticity of reference concrete, the specimen with a replacement ratio of 30% was reduced to 3.7% compared to that of reference concrete, whereas the specimen with a replacement of 100% was reduced 18.5% compared to that of reference concrete. For RFAC specimens, the modulus of elasticity of fiber-reinforced RFAC specimens was higher than that of R-0, except for R-Ny01. Compared with R-0, R-Ny01 has a slightly lower modulus of elasticity. This can be explained by the higher air content of R-Ny01, which can have an effect on the compressive strength and modulus of elasticity of concrete. In addition, the modulus of elasticity was dependent on the density of concrete [31], which was also proved by the reduction in density of R-Ny01 as presented in Table 6.

### 3.4. Splitting Tensile Strength and Flexural Strength

**Figure 6:**
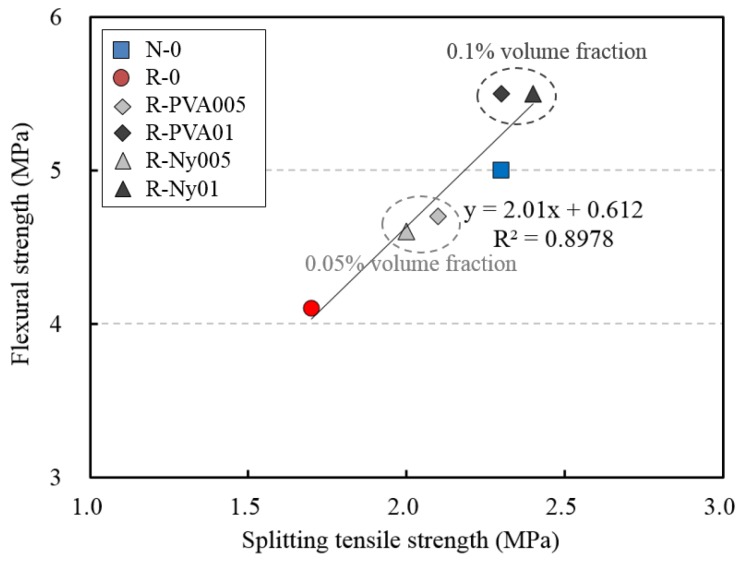
Relationship between splitting tensile strength and flexural strength of concretes.

Table 7 lists the splitting tensile strength and the flexural strength for concrete specimens at 28 days. The splitting tensile strength and the flexural strength of R-0 are 26.1% and 18% lower than those of N-0 specimens, respectively. The higher water absorption of recycled fine aggregate acts to reduce the interfacial bonding capacity between recycled aggregate and cement paste [35]. Also, although fine aggregates are used in saturated surface dry (SSD) conditions, RFAC specimens may contain more water than NFAC specimens due to recycled fine aggregates having about 3.5% higher water absorption compared to natural fine aggregates (Table 1 and Table 2). Thus, it can be inferred that recycled fine aggregates’ characteristics have an influence on the generation of pores in concrete, and result in reduction of its mechanical properties. Generally, the tensile strength of RFAC decreases with an increase in the RFA replacement ratio [36]. The splitting tensile strength and the flexural strength of RFAC specimens were slightly improved when a small volume fraction of synthetic fibers was used. In cases of fiber-reinforced RFAC with a fiber volume fraction of 0.1%, the splitting tensile strength and flexural strength were similar to those of N-0. It can be inferred that the reinforcement of concrete with a small amount of fiber in RFAC contributed to improving the interfacial bonding capacity as compared with R-0. The relationship between the splitting tensile strength and the flexural strength exhibited a comparatively high correlation among all investigated concrete specimens, as shown in Figure 6. It is obvious from the figure that the *R*^2^ value of 0.8978 derived from the regression formula indicates a proportional relationship for the splitting tensile strength and flexural strength in the investigated concrete specimens. The results of regression analysis indicate that flexural strength increases with an increase in the splitting tensile strength.

### 3.5. Shrinkage Cracking Behavior

**Figure 7:**
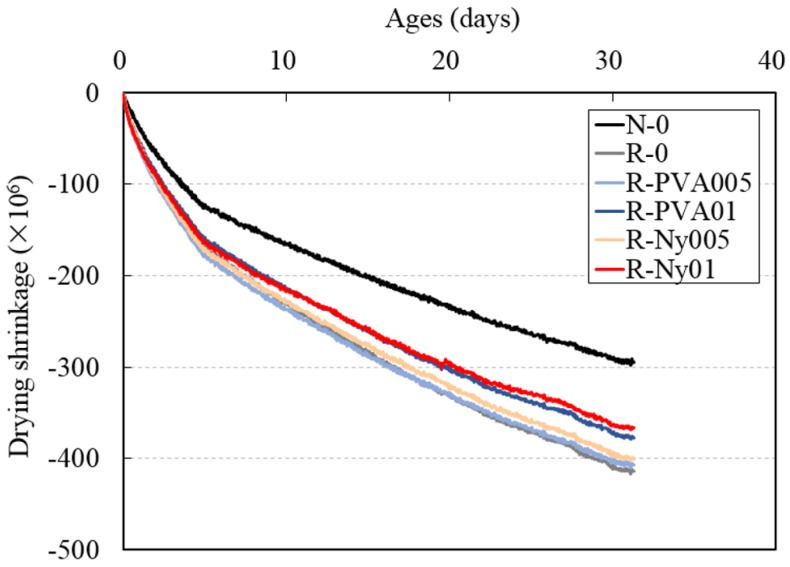
Drying shrinkage of concrete specimens.

To conduct drying shrinkage tests, three replicate samples were prepared and tested at 20 ± 2 °C and 60% ± 5% relative humidity. The results of drying shrinkage tests are shown in Figure 7. It was observed that all the specimens containing RFA had a higher drying shrinkage than N-0, regardless of the fiber reinforcement. This can be attributed to the higher porosity of recycled fine aggregate, which can have an effect on the rapid evaporation of water in concrete [37,38]. For RFAC specimens, the drying shrinkage of fiber-reinforced RFAC specimens was slightly less than that of R-0. Further, the drying shrinkage reduced by increasing the fiber volume fraction. For fiber-reinforced RFAC, the drying shrinkage of the specimens with a 0.1% fiber volume fraction was slightly less than that of the specimens with a 0.05% fiber volume fraction. In addition, the drying shrinkage of nylon fiber-reinforced RFAC was slightly less than that of PVA fiber-reinforced RFAC at the same fiber volume fraction. It is believed that the specific surface between distributed fibers and the concrete matrix for a small volumetric ratio in concrete may influence the drying shrinkage property of concrete. It is well known that the diameter of nylon fiber is about 10 times smaller than that of PVA fiber. Therefore, it can be inferred that the influence on specific surface of nylon fibers in drying shrinkage of the concrete matrix is larger than that of PVA fibers.

**Figure 8:**
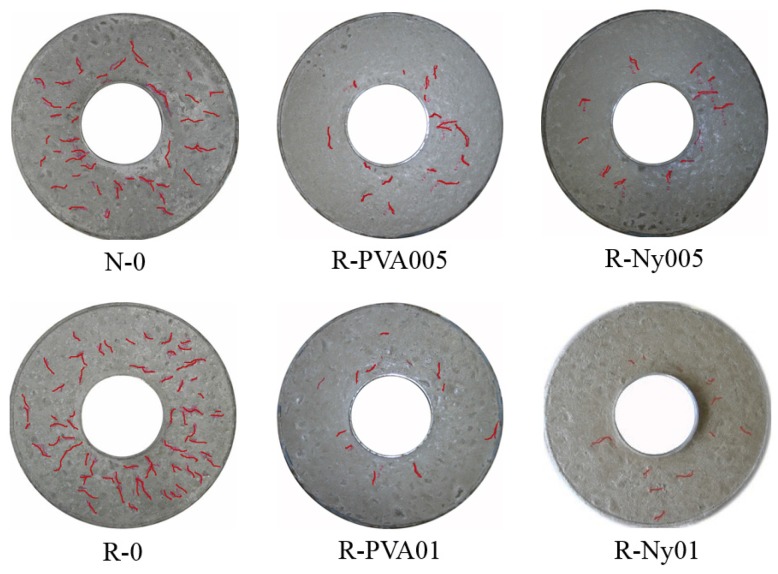
Appearance of surface cracks for concrete in the plat-ring-type test method after 48 h.

**Figure 9:**
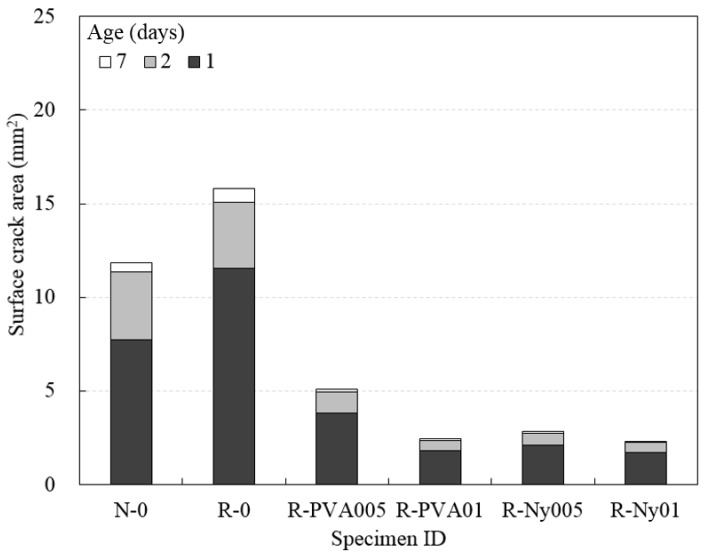
Surface crack area of concrete at tested ages.

Figure 8 shows the surface cracking behavior of concrete specimens in the plat-ring-type test method. The difference in drying shrinkage cracking behavior for concrete specimens was clearly observed via the plat-ring-type tests, with respect to experimental factors such as the type of fine aggregate and fiber reinforcement. Additionally, the surface crack area of concrete specimens in the plat-ring-type test method was measured until seven days, as shown in Figure 9. The crack area was calculated by measuring the width and length of the surface cracks in concrete specimens (Figure 2). It is clear from the figures (Figure 8 and Figure 9) that the surface crack area of R-0 was larger than that of N-0 at all tested ages. Nevertheless, these results of R-0 in terms of cracking behavior were improved by effective reinforcement with PVA and nylon fibers. It can be observed that the propagation of shrinkage cracking in fiber-reinforced RFAC decreased significantly compared to propagation of cracking in N-0 and R-0. Fiber reinforcement with a small volume fraction in RFAC contributed to surface cracking resistance of concrete specimens. In particular, the cracking behaviors of plat-ring-type tests were different from the results by ASTM standard ring tests. Generally, a radial through crack from top to bottom in the concrete specimen was observed from the result of the ASTM standard ring test owing to the crack being induced by the restrained stress of the inner steel ring [39,40]. In this study, however, drying shrinkage cracks were generated on the surface of concrete specimens without through cracks. It is believed that the unpatterned cracks on the surface of the concrete specimens are generated by bleeding and surface drying before the hardened condition. This can be explained by the difference between the plat-ring-type test and the ASTM standard ring test, which is modified in that the thickness and width are thinner and wider than the ASTM standard test specimen of the ring test method.

**Figure 10:**
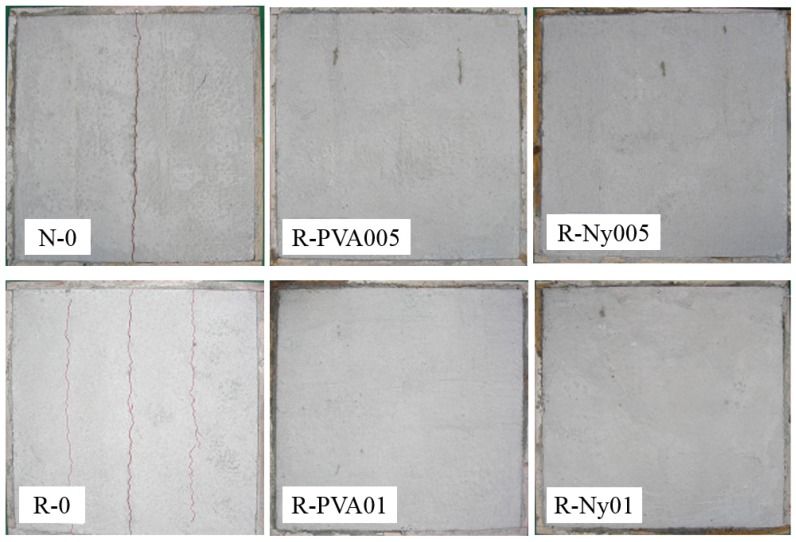
Appearance of surface cracks for concrete in the slab-type test method after outdoor exposure over 16 weeks.

Figure 10 shows the appearance of a surface crack for concrete specimens using the slab-type test method after outdoor exposure for 16 weeks. Additionally, the surface crack area of concrete specimens in the slab-type test method was measured after outdoor exposure for 16 weeks; the results are listed in Table 8. The macro-surface cracks of concrete specimens appeared in the stress risers, which were placed to induce restrained drying shrinkage. The surface crack area for R-0 was 18.9% larger than that for N-0. For non-fiber-reinforced RFAC, the increase in shrinkage cracks depends on the recycled fine aggregate characteristics: not only higher water absorption but also higher porosity. Generally, a higher porosity of recycled fine aggregate degrades the mechanical properties and increases the drying shrinkage [37,38]. However, these shortcomings of RFAC in terms of shrinkage cracking were mitigated by fiber reinforcement. In all the fiber-reinforced RFAC specimens, the surface crack areas were less than 1.24 mm^2^.

Based on the above discussion, we identified that the recycled fine aggregates with 100% replacement influenced the lower mechanical properties and higher shrinkage cracking of concrete. Additionally, it can be concluded that the addition of fibers at a small volume fraction in RFAC is more effective for reducing surface cracks than for improving mechanical performance. In the current study, the surface cracks of RFAC could be controlled by synthetic fiber reinforcement at a small volume fraction of less than 0.1%. In previous studies [41,42], polypropylene fiber reinforcement with small volume fractions such as 0.1%, 0.2% and 0.3% contributed to the reduction in surface crack area or surface crack width in concrete under a drying shrinkage environment. This was attributed to the improvement in the bonding between the cement matrix and the coarse aggregate owing to the addition of fibers. The added fibers affected the resistance of tensile stress generated by surface cracks in fiber-reinforced RFAC. Consequentially, fiber-reinforced RFAC with a small volume fraction does not have a better mechanical performance and lower drying shrinkage compared to those of N-0, as provided by the effectual reduction of surface shrinkage cracks.

## 4. Conclusions

The objective of this study was to experimentally investigate the effectiveness of added fibers at small volume fractions on the mechanical properties and shrinkage cracking of fiber-reinforced RFAC by comparison with NFAC and non-fiber-reinforced RFAC. Based on the experimental results, the main findings of this study, which are useful in describing the mechanical performance and shrinkage cracking capacity control, are as follows:

1. The compressive strength of all RFAC specimens was lower than that of N-0 regardless of fiber reinforcement. This behavior can be attributed to the influence of the weak mechanical bonding between recycled aggregate and cement paste [14,31,32]. The values of compressive strength of the fiber reinforced concretes were not found to be significantly different from the value of the compressive strength of non-reinforced RFAC.
2. The splitting tensile strength and the flexural strength of R-0 were lower than those of N-0 specimens—26.1% and 18%, respectively. However, in cases of fiber-reinforced RFAC with a fiber volume fraction of 0.1%, the splitting tensile strength and flexural strength were similar to those of N-0. It can be inferred that fiber reinforcement of a small amount in RFAC contributed to an improvement in the interfacial bonding capacity between the cement matrix and aggregate.
3. Regardless of the addition of fibers, the drying shrinkage of RFAC was higher than that of N-0. This result can be attributed to the influence of the higher porosity of the recycled fine aggregate [37,38]. This drawback of RFAC for drying shrinkage was improved by the addition of 0.1% PVA and nylon fibers, whose values were lower than the drying shrinkage of R-0 as 8.7% and 11.6% at the tested final ages, respectively. The reason for this behavior can be explained by the improvement in the bond between the cement matrix and coarse aggregate due to added fibers [41,42].
4. The surface crack area of fiber-reinforced RFAC was significantly reduced according to increasing fiber volume fraction compared to that of N-0. These results were observed in both indoor accelerated plat-ring-type tests and long-term outdoor exposure slab-type tests. The results of R-0 in terms of cracking behavior were improved by effective reinforcement with PVA and nylon fibers. Fiber reinforcement with a small volume fraction in RFAC contributed to surface cracking resistance of concrete specimens.
5. It was determined that the effectiveness of added fibers at a small volume fraction in RFAC with 100% fine aggregate replacement is significant for the reduction of surface cracks rather than for improvement of mechanical performance. However, to clarify the effect of fiber reinforcement with a small volume fraction for shrinkage cracking resistance, additional investigations of the influences of the variety of added fiber properties such as aspect ratio, organic or inorganic need to be addressed in future works.

## Figures and Tables

**Table 1:** Material properties.

Materials	Properties
Cement	Ordinary Portland cement
	Density: 3.15 (g/cm^3^)
	Blaine fineness: 3770 (cm^2^/g)
Natural fine aggregate (river sand)	Density: 2.61 (g/cm^3^)
	Water absorption: 1.42 (%)
	Fineness modulus: 2.84
Coarse aggregate	Maximum size: 25 (mm)
	Density: 2.65 (g/cm^3^)
	Water absorption: 1.39 (%)

**Table 2:** Properties of recycled fine aggregate (RFA).

**Property**	**Required by KS F 2573**	**Used**
Density (g/cm^3^)	≥2.2	2.52
Water absorption (%)	≤5.0	4.90
Loss on 0.08 mm sieve passing (%)	≤7.0	3.75
Impurity (%)	≤1.0	0.07
**Sieve Analysis (Passing, %)**	**Standard Range**	**Measured Value**
5 mm	90–100	100
2.5 mm	80–100	99
1.2 mm	50–90	86
0.6 mm	25–65	65
0.3 mm	10–35	33
0.15 mm	2–15	12

**Table 3:** Fiber properties.

Fiber Type	Specific Density (g/cm^3^)	Length (mm)	Diameter (μm)	Tensile Strength (MPa)	Elastic Modulus (GPa)
PVA	1.30	12	200	910	29
Nylon	1.16	19	23	896	5.17

**Table 4:** Mixture proportions of concrete specimens.

ID ^a^	W/C ^b^ (%)	Cement (kg/m^3^)	S/A ^c^ (%)	Water (kg/m^3^)	Coarse Aggregate (kg/m^3^)	NFA ^d^ (kg/m^3^)	RFA (kg/m^3^)	Fiber (V*_f_* %) ^e^	SP ^f^ (kg/m^3^)
N-0	50	352	47	176	944	809	-	-	0.7
R-0	50	352	47	176	944	-	727	-	0.7
R-PVA005	50	352	47	176	944	-	727	0.05	0.7
R-PVA01	50	352	47	176	944	-	727	0.1	1.4
R-Ny005	50	352	47	176	944	-	727	0.05	1.4
R-Ny01	50	352	47	176	944	-	727	0.1	1.8

^a^ Type of fine aggregate, type of added fibers, and fiber volume fraction; ^b^ Water-cement ratio; ^c^ Sand-coarse aggregate ratio; ^d^ Natural fine aggregate; ^e^ Fiber volume fraction; ^f^ Superplasticizer.

**Table 5:** Properties of fresh concrete for each mixture.

ID	Slump (mm) ^a^	Air Content (%)	SP (kg/m^3^)
N-0	200	3.2	0.7
R-0	184	3.5	0.7
R-PVA005	175	5.6	0.7
R-PVA01	165	5.5	1.4
R-Ny005	165	6.0	1.4
R-Ny01	185	7.5	1.8

^a^ Target value range of slump: 180 ± 20 mm.

**Table 6:** Density of concrete.

Specimen ID	Density (kg/m^3^)
N-0	2709
R-0	2591
R-PVA005	2606
R-PVA01	2556
R-Ny005	2560
R-Ny01	2486

**Table 7:** Splitting tensile strength and flexural strength of concrete specimens.

ID	Splitting Tensile Strength (MPa) ^a^	Flexural Strength (MPa) ^a^
N-0	2.3	5.0
R-0	1.7	4.1
R-PVA005	2.1	4.7
R-PVA01	2.3	5.5
R-Ny005	2.0	4.6
R-Ny01	2.4	5.5

^a^ Average value of three replicate samples at 28 days.

**Table 8:** Surface crack area of concrete after outdoor exposure over 16 weeks in the slab-type test.

Specimen ID	Surface Crack Area (mm^2^)
N-0	35.86
R-0	42.64
R-PVA005	1.24
R-PVA01	0.70
R-Ny005	1.17
R-Ny01	0.68
